# Extracting Cardiorespiratory Symptoms From Clinical Notes Using Open-Weight Large Language Models: Method Development and Validation Study

**DOI:** 10.2196/89480

**Published:** 2026-05-22

**Authors:** Yunbing Bai, Wanting Cui, Joseph Finkelstein

**Affiliations:** 1 Arizona Center for Telemedicine and Digital Health, College of Medicine, University of Arizona, 1501 N Campbell Ave AHSL 1156, Tucson, AZ, 85724-5105, United States, 1 520-626-3944

**Keywords:** natural language processing, NLP, large language models, electronic health records, signs and symptoms, clinical coding, prompt engineering, named entity recognition

## Abstract

**Background:**

Accurate identification of clinical symptoms and signs (S&S) is essential for the early detection of high-burden cardiorespiratory conditions, including lung cancer, chronic obstructive pulmonary disease, and heart failure. Although symptom data play a central role in diagnostic reasoning and predictive modeling, most S&S information remains embedded in unstructured electronic health record notes, limiting their use in automated phenotyping, surveillance, and clinical decision support. Traditional natural language processing systems struggle with domain variability and contextual nuance in clinical text. Recent advances in large language models (LLMs) offer a promising alternative, yet challenges remain in hallucinations, overinference, and safe deployment. This study evaluated whether locally deployed open-source models could reliably extract cardiorespiratory S&S and map them to *ICD-10-CM* (*International Classification of Diseases, Tenth Revision, Clinical Modification*) codes using optimized prompting strategies.

**Objective:**

This study aims to assess the accuracy of open-source LLMs in extracting explicitly stated cardiorespiratory S&S from clinical notes and mapping them to *ICD-10-CM* codes (R00-R09) and to compare performance across 4 prompt-engineering strategies, including a multimodule LLM framework.

**Methods:**

A total of 593 clinical notes from the MTSamples database were manually reviewed, with 93 notes used for prompt development and comparison using Llama 3.3-70B, and 500 notes used as testing data for the final best prompt setting using both Llama 3.3-70B and gpt-oss-120B. Four prompting conditions were evaluated: (1) instruction-only, (2) *ICD-10-CM* definition–based prompts, (3) assumption-free prompts, and (4) a multimodule LLM framework with postprocessing. Performance was measured using precision, recall, and *F*_1_-score for both S&S extraction and *ICD-10-CM* code generation.

**Results:**

Across all prompt strategies, model performance improved as more structure and constraints were added. Instruction-only prompting demonstrated high recall but poor precision. Incorporating *ICD-10-CM* definitions improved coding accuracy, and assumption-free prompting further balanced precision and recall. The multimodule approach with postprocessing achieved the highest performance during prompt development. On the independent test corpus, entity-level microaveraged evaluation showed that gpt-oss-120B outperformed Llama 3.3-70B in both tasks. For S&S extraction, Llama 3.3-70B achieved a precision of 0.63, a recall of 0.86, and an *F*_1_-score of 0.73, whereas gpt-oss-120B achieved a precision of 0.89, a recall of 0.87, and an *F*_1_-score of 0.88. For *ICD-10-CM* code mapping, Llama 3.3-70B achieved a precision of 0.59, a recall of 0.83, and an *F*_1_-score of 0.69, whereas gpt-oss-120B achieved a precision of 0.90, a recall of 0.84, and an *F*_1_-score of 0.87.

**Conclusions:**

Locally deployed LLMs, when paired with optimized prompting and multimodule orchestration, can accurately extract cardiorespiratory S&S and generate *ICD-10-CM* codes from unstructured clinical notes. This approach increases the level of data safety by enabling on-premises processing without external data transmission and demonstrates strong potential for scalable, domain-adaptive symptom extraction pipelines in biomedical informatics. Future work should expand datasets and evaluate generalizability across clinical domains.

## Introduction

Understanding the clinical symptoms and signs (S&S) of high-burden cardiorespiratory conditions—such as lung cancer, chronic obstructive pulmonary disease (COPD), and heart failure—is essential for timely diagnosis, risk prediction, and improved patient outcomes. For example, persistent cough, dyspnea, chest discomfort, or hemoptysis can expedite lung cancer detection, allowing treatment at earlier stages when prognosis is more favorable [1-3]. In heart failure, monitoring subtle changes in breathing patterns, exercise tolerance, or peripheral edema supports the early prediction of disease onset and the prevention of acute decompensations [4]. Similarly, the early identification of worsening respiratory symptoms—such as increased breathlessness or sputum production—can help predict and prevent COPD exacerbations, a leading cause of hospitalization and mortality [5].

In clinical practice, symptom data play a pivotal role in both diagnostic decision-making and predictive modeling. Hospital readmission risk models often incorporate structured and unstructured symptom information to identify patients at higher risk for rehospitalization [6]. Predictive algorithms for heart failure onset and COPD exacerbation frequently rely on longitudinal patterns in clinical data—including symptom trajectories—to generate early alerts for clinicians [4,7,8]. Likewise, lung cancer diagnostic pathways integrate symptom reports with imaging and laboratory findings to guide further evaluation [1,3].

Despite their importance, most S&S are documented in unstructured free-text clinical notes rather than structured electronic health record fields. These unstructured data contain nuanced clinical observations valuable for predicting lung cancer, heart failure onset, COPD exacerbation, and hospital readmission risk. However, the complexity, variability, and domain-specific language of clinical text pose significant challenges for traditional natural language processing (NLP) methods [6-8]. Advances in large language models (LLMs) have shown promise in overcoming these challenges, enabling more accurate and timely extraction of clinically relevant information across diverse documentation styles and supporting predictive analytics in high-impact conditions [9,10].

To address these challenges, specialized biomedical NLP models such as BioBERT and BioRAG have been developed to increase accuracy in symptom identification [6-8,11,12]. These models are particularly effective in detecting symptoms associated with chronic conditions and cancers, making them valuable tools for early disease prediction. However, their dependency on task-specific training datasets can restrict adaptability to new medical contexts. Advanced generative models, such as GPT-4o, have shown promise in identifying symptoms across clinical domains and require little pretraining [9,13]. However, most models are for general purposes that are not fully suitable for medical tasks. In addition, while these models are convenient to use, they often run on commercial cloud platforms, which can raise serious privacy concerns when handling sensitive patient data.

In the *ICD-10-CM* (*International Classification of Diseases, Tenth Revision, Clinical Modification*) coding system, there is a dedicated section for S&S, ranging from R00 to R99 (symptoms, signs, and abnormal clinical and laboratory findings). Our previous studies showed that some LLMs, capable of being deployed and operated locally, demonstrated strong performance in extracting S&S and mapping them to *ICD-10-CM* codes within the genitourinary system, specifically in the range of R30 to R39 (symptoms and signs involving the genitourinary system) [14,15]. However, as task complexity increased, model performance became unstable. In this study, we aim to explore the feasibility of using LLMs with different prompt-engineering strategies to accurately extract cardiorespiratory-related S&S from medical text, map the extracted S&S to standardized *ICD-10-CM* codes in the range of R00 to R09 (symptoms and signs involving the circulatory and respiratory systems), and generate structured outputs to support automated evaluation.

## Methods

### Data Source

This study conducted a comprehensive analysis of clinical notes sourced from the MTSamples database [16], with a specific focus on records related to medical conditions. The initial phase involved identifying notes that contained keywords associated with cardiorespiratory disorders. These notes underwent manual review, during which any entries that did not explicitly describe clinical S&S were excluded. After this process, a final dataset comprising 93 notes was selected. These notes were retained in their original form, without any textual modifications. For evaluation purposes, annotations provided by clinical experts served as the gold standard. We only extracted and annotated S&S with *ICD-10-CM* codes related to the cardiorespiratory system that were able to map to *ICD-10-CM* codes: R00 to R09 [17]. These 93 cardiorespiratory clinical notes served as the training data for prompt development.

An additional 500 expert-labeled clinical notes from the MTSamples database were used as the test dataset. Unlike the training data, the test notes were not limited to the cardiorespiratory domain; instead, they included S&S spanning multiple clinical systems. This design more closely reflects real-world clinical documentation and enables a more realistic evaluation of model performance.

Each clinical note was annotated by 2 individuals including a nurse and a clinical informatician with experience in medical coding. Cohen κ coefficient was used to evaluate the agreement between 2 annotators as previously described. The interrater reliability based on the Cohen κ coefficient was 0.75 (SD 0.06) in this study.

### Prompt Engineering and LLM

In this study, we selected 2 open-weight LLMs for evaluation: Llama 3.3-70B and gpt-oss-120B. Prompt development was conducted using Llama 3.3-70B for iterative prompt engineering and method design [18]. We chose Llama 3.3-70B based on our previous studies, in which it demonstrated strong performance in extracting clinical S&S from medical notes [14,15].

After the final prompt framework was established, we evaluated the proposed approach using both Llama 3.3-70B and gpt-oss-120B to assess whether the workflow generalized across different open-weight models [19]. For both models, the temperature was set to 0, and all other hyperparameters were kept at their default settings during evaluation.

We conducted all experiments on a local workstation equipped with a 32-core 2.50 GHz Intel Xeon W7-3565X processor, 256 GB of RAM, and 2 NVIDIA RTX PRO 6000 Blackwell Max-Q graphics cards with a total of 192 GB of graphics processing unit memory. Both models were deployed locally using Ollama, a lightweight and extensible framework for local LLM deployment. Llama 3.3-70B was run using Q4_K_M quantization, whereas gpt-oss-120B was run using MXFP4 quantization [18,19]. No central processing unit offloading was used during inference. The approximate inference speed was 27.88 tokens/s for Llama 3.3-70B and 152.73 tokens/s for gpt-oss-120B. Although gpt-oss-120B has a larger total parameter count than Llama 3.3-70B, its faster throughput on the same hardware is not necessarily unexpected because gpt-oss-120B is a sparse mixture-of-experts model, meaning that only a subset of parameters is active for each token during inference. By contrast, Llama 3.3-70B is a dense model. The difference may also reflect the deployment formats used in this study, as gpt-oss-120B was run in MXFP4 and Llama 3.3-70B in Q4_K_M.

The prompt was designed to accomplish 2 primary tasks: extracting the exact S&S from clinical notes and accurately mapping the extracted S&S to the correct *ICD-10-CM* code groups (R00-R09). To achieve these objectives, multiple prompt structures were tested before selecting an instruction-based prompting approach. The prompt template we applied organized the prompt into 3 distinct sections: “TASK,” “REQUIREMENT,” and “CLINICAL NOTE.” The TASK section clearly defined the objective, instructing the model to extract S&S explicitly mentioned in the clinical notes and associate them with the correct *ICD-10-CM* codes. The REQUIREMENT section provided specific constraints to guide the extraction process. The CLINICAL NOTE section contained the original medical transcription text, from which the model was expected to extract relevant information.

**Figure 1.**
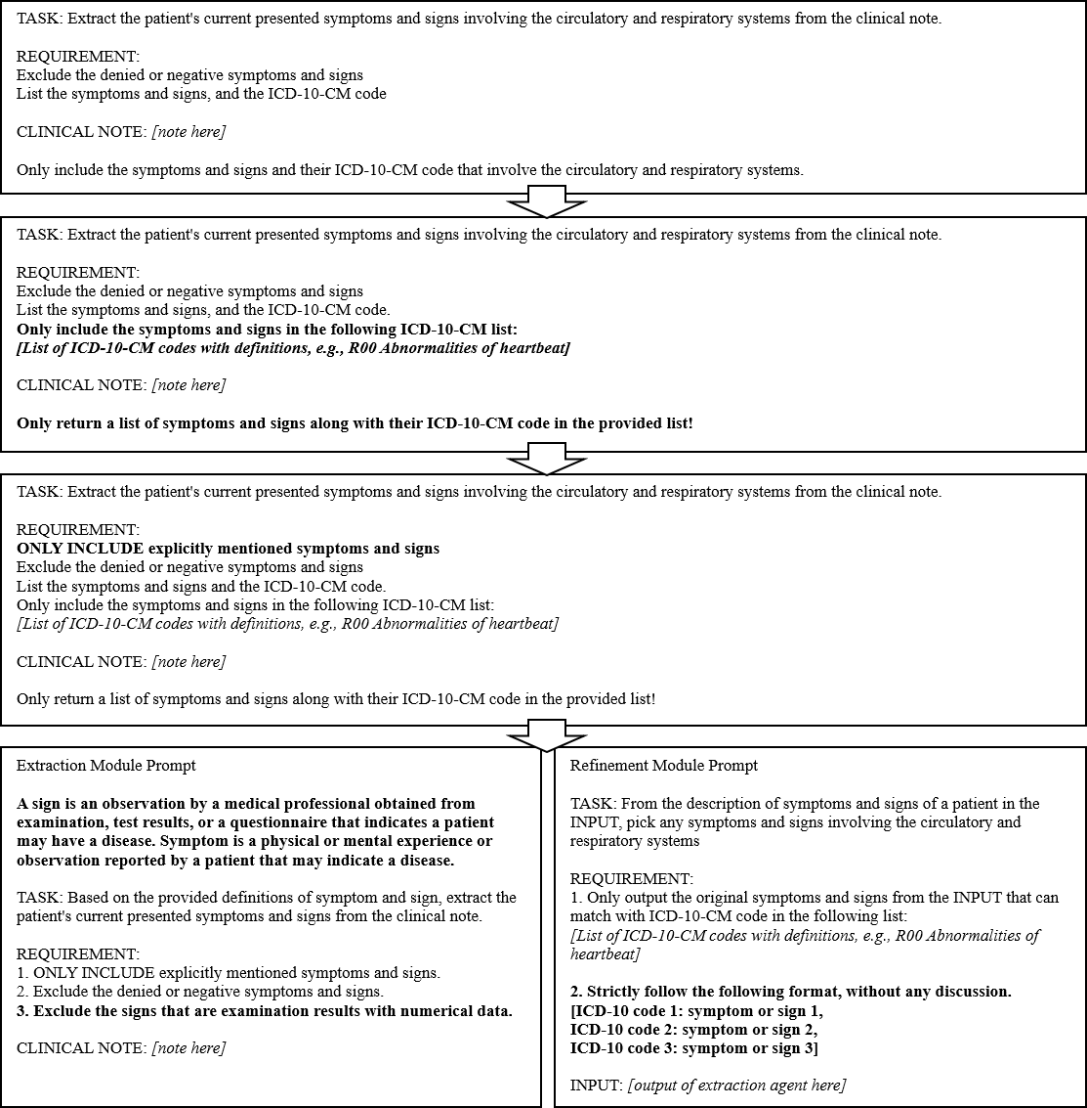
Overview of the 4 prompt-engineering iterations evaluated in this retrospective methodological study for extracting cardiorespiratory symptoms and signs from unstructured clinical notes. The study used deidentified medical transcription notes from the publicly available MTSamples database. Iterations progressed from instruction-only prompting to *ICD-10-CM* (*International Classification of Diseases, Tenth Revision, Clinical Modification*) definition–based prompts, assumption-free constraints, and finally a multimodule large language model framework with role separation (extraction module and refinement module). Each iteration introduced additional constraints to reduce hallucinations, limit inference, and improve structured *ICD-10-CM* code generation (R00-R09). Bolded elements indicate newly added prompt components at each iteration.

We iterated through 4 generations of prompts, aiming to improve the model’s extraction of clinical S&S, as shown in Figure 1. In our initial approach, we provided the task background and goals within the prompt to evaluate the feasibility and general performance of the model without further instructions on extracting S&S or converting them to *ICD-10-CM* codes.

Then, in the second generation, we provided the model with a list of *ICD-10-CM* codes and their definitions within the prompt. To balance the need for completeness while controlling the input length, we limited the *ICD-10-CM* codes to those within the range R00 to R09, specifically including 3-character and 4-character codes (R00-R09 and R00.0-R09.9). These codes corresponded to “symptoms and signs involving the circulatory and respiratory systems” (Table 1). In addition, clinical notes often contained negative S&S, such as “No cough, shortness of breath, fever, or chills.” To ensure that only positively reported S&S were extracted, we explicitly instructed the model to exclude any negative S&S from its output. In the third iteration, we aimed to reduce hallucinations by explicitly instructing the model to extract only S&S explicitly mentioned in the clinical notes and to avoid making assumptions about content not explicitly stated.

**Table 1.** *ICD-10-CM*^a^ symptom and sign codes (R00-R09) and corresponding definitions related to the circulatory and respiratory systems that were explicitly provided to the large language models during prompt engineering^b^.

*ICD-10-CM* code	Definition
R00	Abnormalities of heart beat
R00.0	Tachycardia, unspecified
R00.1	Bradycardia, unspecified
R00.2	Palpitations
R00.8	Other abnormalities of heart beat
R00.9	Unspecified abnormalities of heart beat
R01	Cardiac murmurs and other cardiac sounds
R01.0	Benign and innocent cardiac murmurs
R01.1	Cardiac murmur, unspecified
R01.2	Other cardiac sounds
R03	Abnormal blood pressure reading, without diagnosis
R03.0	Elevated blood pressure reading, without diagnosis of hypertension
R03.1	Nonspecific low blood pressure reading
R04	Hemorrhage from respiratory passages
R04.0	Epistaxis
R04.1	Hemorrhage from throat
R04.2	Hemoptysis
R04.8	Hemorrhage from other sites in respiratory passages
R04.9	Hemorrhage from respiratory passages, unspecified
R05	Cough
R05.1	Acute cough
R05.2	Subacute cough
R05.3	Chronic cough
R05.4	Cough syncope
R05.8	Other specified cough
R05.9	Cough, unspecified
R06	Abnormalities of breathing
R06.0	Dyspnea
R06.1	Stridor
R06.2	Wheezing
R06.3	Periodic breathing
R06.4	Hyperventilation
R06.5	Mouth breathing
R06.6	Hiccough
R06.7	Sneezing
R06.8	Other abnormalities of breathing
R06.9	Unspecified abnormalities of breathing
R07	Pain in throat and chest
R07.0	Pain in throat
R07.1	Chest pain on breathing
R07.2	Precordial pain
R07.8	Other chest pain
R07.9	Chest pain, unspecified
R09	Other symptoms and signs involving the circulatory and respiratory system
R09.0	Asphyxia and hypoxemia
R09.1	Pleurisy
R09.2	Respiratory arrest
R09.3	Abnormal sputum
R09.8	Other specified symptoms and signs involving the circulatory and respiratory systems
R09.A	Foreign body sensation of the circulatory and respiratory system

a *ICD-10-CM*: *International Classification of Diseases, Tenth Revision, Clinical Modification*.

b These codes represent standardized clinical symptom categories used for mapping extracted symptoms and signs in a retrospective analysis of deidentified clinical notes from the MTSamples database. Only 3-character and 4-character *ICD-10-CM* codes within the R00 to R09 range were included to limit the input length of models.

In the fourth iteration, we aimed to prompt the model to produce structured responses that would enable automated evaluation. However, to avoid increasing task complexity and causing unstable outputs, we adopted a multimodule LLM approach in which specialized modules (eg, extraction and refinement) collaborate through role-based interactions to decompose the task and generate standardized outputs—an approach that has been shown to improve reliability and orchestration in complex workflows [20-23].

Specifically, we used 2 modules: an extraction module (EM) and a refinement module (RM). The EM was responsible for identifying all valid S&S explicitly mentioned in the clinical notes. This task consisted of two subtasks: (1) extracting candidate S&S and (2) filtering out false positives. The definitions of S&S from the National Cancer Institute were provided in the prompt, and the model was instructed to include only explicitly stated S&S and to exclude negated or denied findings. During preliminary testing, the model occasionally interpreted examination results—such as blood pressure values—as clinical signs. To mitigate this issue, we further instructed the model to exclude examination findings that contained numerical values.

The RM was responsible for mapping the extracted S&S to *ICD-10-CM* codes in the range R00 to R09. For this module, we provided a predefined list of *ICD-10-CM* codes within this range, along with the required output format. The RM ingested the unstructured plain text output generated by the EM, selected S&S that matched the provided code list, and returned the results in the specified format.

Following model inference, we performed postprocessing and data cleaning on the RM’s structured outputs to eliminate assumption-based results. Based on a review of outputs from earlier iterations, we developed a red-flag keyword list (Textbox 1). Any output entries containing terms from this list were flagged as high risk for false positives due to assumptions, inclusion of negated S&S, or hallucinations. All such entries were subsequently removed from the final results.

Textbox 1. **Red-flag keyword list used during postprocessing to identify and remove high-risk false-positive outputs generated by the multimodule large language model (LLM) framework. Keywords reflect assumption-based language, negation, inference, or nonexplicit symptom reporting. Any extracted symptom or *ICD-10-CM* (*International Classification of Diseases, Tenth Revision, Clinical Modification*) code description containing one or more of these terms was excluded from final results.**

**Red-flag words**

- Possible
- No
- Related
- Mentioned
- Not present
- Appears
- Implied
- Normal
- Considered
- Involving
- Explicitly

### Evaluation

The model’s performance was assessed through a 2-part evaluation for each clinical note. First, the accuracy of extracted S&S was measured to determine how well the model identified only the explicitly stated S&S from the text. Second, the accuracy of generated *ICD-10-CM* codes was evaluated to assess the model’s ability to correctly map extracted S&S to the appropriate *ICD-10-CM* categories.

To quantify performance, precision, recall, and *F*_1_-score were used, with human expert annotations serving as the gold standard for comparison. Precision calculated how many predicted S&S were correct, and recall calculated how many ground-truth S&S were recognized. Model outputs were evaluated at the individual clinical note level, and average performance metrics were then calculated across all notes. For *ICD-10-CM* code evaluation, only the first 3 digits of the code were considered to account for variations in granularity. For example, R01, R01.1, and R01.21 were all treated as R01, ensuring consistency in assessment.


$$Precision=\;\frac{True\;Positive}{True\;Positive+False\;Positive}$$

$$Recall=\;\frac{True\;Positive}{True\;Positive+False\;Negative}$$

$$F_{1}-score\;=2\times \frac{Precision\times Recall}{Precision+Recall}$$

Prompt development was conducted using Llama 3.3-70B. Accordingly, performance on the prompt development dataset was compared across prompt iterations to assess how changes in prompt design influenced model outputs. To reduce the risk of overfitting the final framework to either the development data or the model used for prompt engineering, both Llama 3.3-70B and gpt-oss-120B were evaluated on the independent test dataset.

Because the test dataset included notes from multiple clinical systems rather than only cardiorespiratory conditions, many notes contained no annotated cardiorespiratory S&S. In an initial note-level evaluation, notes with both empty ground-truth labels and empty model outputs were treated as correct matches. However, because this macro-averaging approach may inflate overall performance in a sparse dataset, we additionally calculated precision, recall, and *F*_1_-score using standard entity-level microaveraging across the corpus.

For microaveraged evaluation, predicted and reference labels were first reduced to unique sets within each clinical note. True positives were defined as labels present in both the model output and expert annotation for the same note, false positives as labels predicted by the model but absent from the reference set, and false negatives as reference labels missed by the model. These counts were then summed across all notes, and precision, recall, and *F*_1_-score were calculated from the aggregated totals. For *ICD-10-CM* code evaluation, only the first 3 characters of each code were used in this corpus-level analysis. Notes with no gold labels and no predicted labels did not contribute to true-positive counts and were not assigned perfect scores.

In addition to reporting overall performance, we evaluated the model separately for each primary *ICD-10-CM* category within the R00 to R09 range. For this subgroup analysis, both predicted and reference codes were grouped at the 3-character category level (eg, R00, R01, R03, R04, R05, R06, R07, and R09), and precision, recall, and *F*_1_-score were calculated independently for each category. Within each category, true positives represented correctly predicted codes that matched the expert annotation for the same clinical note, false positives represented incorrectly predicted codes assigned to that category, and false negatives represented annotated codes in that category that were not identified by the model. This analysis was included to characterize variation in performance across individual cardiorespiratory *ICD-10-CM* subcategories.

### Ethical Considerations

This study did not involve human participants or the collection of identifiable private information. All analyses were conducted using secondary data from the publicly available MTSamples database, which consists of deidentified clinical transcription samples created for educational and research purposes [16]. No patient identifiers were accessed, recorded, or analyzed, and no attempt was made to reidentify individuals. In accordance with journal guidelines, this research does not meet the definition of human subjects research and was therefore exempt from institutional review board review and informed consent requirements. The study was conducted in compliance with applicable ethical standards for research using deidentified secondary data.

## Results

Among the 93 human-annotated clinical notes, a total of 168 S&S related to the cardiorespiratory system were identified. On average, each note contained 1.81 (SD 1.09) labeled symptoms. The most frequently observed *ICD-10-CM* category was R06—Abnormalities of breathing, present in 52 notes, followed by R01—Cardiac murmurs and other cardiac sounds (31 notes), R00—Abnormalities of heart beat (26 notes), and R07—Pain in throat and chest (25 notes) [17].

Performance varied across the 4 prompt settings evaluated (Table 2). The instruction-only prompt resulted in high recall but low precision in both tasks, yielding an *F*_1_-score of 0.54 for S&S extraction and 0.41 for *ICD-10-CM* code generation. Introducing *ICD-10-CM* definitions improved both precision and *F*_1_-scores, especially for code generation (*F*_1_-score=0.70), though a slight reduction in recall was observed in extraction. Further improvement was seen when assumption-free constraints were added, increasing *F*_1_-scores to 0.69 for extraction and 0.74 for code generation, with more balanced precision-recall trade-offs.

**Table 2.** Performance comparison of 4 prompt-engineering strategies for extracting cardiorespiratory symptoms and signs and generating corresponding *ICD-10-CM*^a^ codes (R00-R09).

Prompt setting	Precision	Recall	*F*_1_-score^b^
S&S^c^ extraction			
Instruction only	0.38	0.92	0.54
*ICD-10-CM* code–based symptom extraction	0.51	0.86	0.64
Assumption-free constraints	0.57	0.86	0.69
Multimodule LLMs^d^	0.80	0.94	0.86
Multimodule LLMs with postdata cleaning	0.86	0.94	0.90
*ICD-10-CM* code generation			
Instruction only	0.29	0.71	0.41
*ICD-10-CM* code–based symptom extraction	0.58	0.89	0.70
Assumption-free constraints	0.69	0.80	0.74
Multimodule LLMs	0.79	0.96	0.87
Multimodule LLMs with postdata cleaning	0.83	0.95	0.89

a *ICD-10-CM*: *International Classification of Diseases, Tenth Revision, Clinical Modification*.

b Precision, recall, and *F*_1_-score were calculated at the clinical note level and averaged across notes. The results are shown separately for S&S extraction and *ICD-10-CM* code generation, demonstrating progressive performance improvements, as additional constraints and a multimodule architecture with postprocessing were introduced.

c S&S: symptom and sign.

d LLM: large language model.

The highest performance was achieved using a multimodule LLM framework with postdata cleaning. This setting produced an *F*_1_-score of 0.90 for S&S extraction and 0.89 for *ICD-10-CM* code generation, reflecting strong gains in both precision and recall. These results demonstrate the progressive effectiveness of structured prompting and refinement strategies in improving model outputs across clinical NLP tasks.

Overall, the multimodule architecture demonstrated superior performance. Without postprocessing, the model achieved an *F*_1_-score of 0.86 for S&S extraction and 0.87 for *ICD-10-CM* code generation, indicating substantial improvements in both precision and recall. However, the model occasionally produced unstable outputs containing assumption-based errors. Incorporating postprocessing and data-cleaning steps eliminated these assumptions from the multimodule outputs and further improved performance, yielding *F*_1_-scores of 0.90 for S&S extraction and 0.89 for *ICD-10-CM* code generation.

In the test dataset, a total of 1601 S&S were labeled. On average, each clinical note contained 3.20 (SD 2.24) labeled S&S. Among these notes, 69 contained at least one cardiorespiratory symptom or sign, with an average of 1.33 (SD 0.70) cardiorespiratory S&S per note. The multimodule LLM framework with postprocessing was evaluated on this dataset using both Llama 3.3-70B and gpt-oss-120B (Table 3).

**Table 3.** Evaluation results of the proposed multimodule large language model framework with postprocessing data cleaning for extracting cardiorespiratory symptoms and signs (S&S) and generating corresponding *ICD-10-CM*^a^ codes (R00-R09)^b^.

Data and model		Precision	Recall	*F*_1_-score
All testing clinical notes (n=500)				
S&S^c^ extraction				
Llama 3.3-70B		0.93	0.97	0.95
gpt-oss-120B		0.98	0.97	0.97
*ICD-10-CM* code generation				
Llama 3.3-70B		0.94	0.98	0.95
gpt-oss-120B		0.98	0.98	0.98
Testing notes with cardiorespiratory S&S (n=69)				
S&S extraction				
Llama 3.3-70B		0.89	0.83	0.86
gpt-oss-120B		0.97	0.86	0.91
*ICD-10-CM* code generation				
Llama 3.3-70B		0.90	0.85	0.87
gpt-oss-120B		0.97	0.87	0.92

a *ICD-10-CM*: *International Classification of Diseases, Tenth Revision, Clinical Modification*.

b This retrospective evaluation was conducted using an independent test dataset of 500 deidentified clinical notes from the publicly available MTSamples database. The test dataset included notes spanning multiple clinical systems, of which a subset contained cardiorespiratory symptoms. Performance is reported separately for (1) all test notes (n=500) and (2) notes containing at least one cardiorespiratory S&S (n=69). Two locally deployable, open-weight large language models—Llama 3.3-70B and gpt-oss-120B—were evaluated using identical multimodule workflows and postprocessing rules. Precision, recall, and *F*_1_-score were calculated at the clinical note level and macroaveraged across notes. This table demonstrates the robustness and cross-model generalizability of the proposed strategy for cardiorespiratory symptom extraction and *ICD-10-CM* mapping.

c S&S: symptoms and signs.

For Llama 3.3-70B, performance on notes containing cardiorespiratory S&S was comparable to that observed in the prompt development dataset. gpt-oss-120B, which is a more recent model with a larger number of parameters than Llama 3.3-70B, achieved overall better performance. This result demonstrates the strong generalizability of the proposed multimodule LLM framework across different model architectures.

Under standard entity-level microaveraging across the full test corpus (Table 4), performance was lower than in the note-level evaluation. gpt-oss-120B outperformed Llama 3.3-70B in both tasks, achieving an *F*_1_-score of 0.88 for S&S extraction and 0.87 for *ICD-10-CM* code generation, compared with 0.73 and 0.69, respectively, for Llama 3.3-70B. These findings indicate that although note-level results were high, corpus-level microaveraged evaluation provided a more conservative estimate of extraction and coding performance.

**Table 4.** Standard entity-level microaveraged performance across the independent test corpus (n=500)^a^.

Model	Precision	Recall	*F*_1_-score
S&S^b^ extraction			
Llama 3.3-70B	0.63	0.86	0.73
gpt-oss-120B	0.89	0.87	0.88
*ICD-10-CM*^c^ code generation			
Llama 3.3-70B	0.59	0.83	0.69
gpt-oss-120B	0.90	0.84	0.87

a Precision, recall, and *F*_1_-score were calculated by summing true positives, false positives, and false negatives across all notes after reducing predicted and reference labels to unique sets within each note. The results are shown separately for symptoms and signs extraction and *ICD-10-CM* code generation for Llama 3.3-70B and gpt-oss-120B. *ICD-10-CM* codes were evaluated at the 3-character category level.

b S&S: symptoms and signs.

c *ICD-10-CM*: *International Classification of Diseases, Tenth Revision, Clinical Modification*.

Category-specific analysis (Table 5) showed that model performance varied across individual *ICD-10-CM* groups. For gpt-oss-120B, the highest *F*_1_-scores were observed for R05 (cough; *F*_1_-score=1.00), R06 (abnormalities of breathing; *F*_1_-score=0.96), and R04 (hemorrhage from respiratory passages; *F*_1_-score=0.94), whereas lower performance was seen for R09 (other circulatory and respiratory symptoms and signs; F1=0.71) and R03 (abnormal blood-pressure reading; *F*_1_-score=0.73). Llama 3.3-70B showed a similar pattern of stronger performance in more common and clinically explicit categories such as R06 (*F*_1_-score=0.83) and R04 (*F*_1_-score=0.80), but lower performance in R03 (*F*_1_-score=0.33) and R01 (cardiac murmurs and other cardiac sounds; *F*_1_-score=0.40). These results suggest that model accuracy was higher for more explicit and frequently occurring symptom categories, while broader or less frequently represented categories remained more challenging.

For Llama 3.3-70B, 452 (90.4%) of 500 test clinical notes were correctly matched to the expert labels, leaving 48 (9.6%) notes with at least 1 error. Among these 48 error notes, 26 (54.2%) contained hallucinated S&S, 10 (20.8%) included S&S or diagnoses from noncardiorespiratory systems that were incorrectly identified as cardiorespiratory S&S, and 14 (29.2%) missed at least 1 true cardiorespiratory S&S present in the clinical note. These error categories were not mutually exclusive, and some notes contained more than 1 error type. For gpt-oss-120B, 477 (95.4%) of 500 test clinical notes were correctly matched to the expert labels, leaving 23 (4.6%) notes with at least 1 error. Among these 23 error notes, 4 (17.4%) contained hallucinated S&S, 7 (30.4%) included S&S or diagnoses from noncardiorespiratory systems that were incorrectly identified as cardiorespiratory S&S, and 12 (52.2%) missed at least 1 true cardiorespiratory S&S present in the clinical note. As with Llama 3.3-70B, these categories were not mutually exclusive. The most frequent errors reflected assumption-based interpretation, particularly inferring increased or decreased blood pressure from numerical blood pressure readings alone.

**Table 5.** Category-specific *ICD-10-CM* code generation performance for each included 3-character cardiorespiratory category in the independent test dataset^a^.

*ICD-10-CM*^b^ category	Llama 3.3-70B			gpt-oss-120B		
	Precision	Recall	*F*_1_-score	Precision	Recall	*F*_1_-score
R00	0.42	0.89	0.57	0.75	1.00	0.86
R01	0.33	0.50	0.40	0.67	1.00	0.80
R03	0.40	0.29	0.33	1.00	0.57	0.73
R04	0.73	0.89	0.80	1.00	0.89	0.94
R05	0.75	0.75	0.75	1.00	1.00	1.00
R06	0.70	1.00	0.83	0.96	0.96	0.96
R07	0.53	0.90	0.67	0.77	1.00	0.87
R09	0.47	0.78	0.58	0.85	0.61	0.71

a Precision, recall, and *F*_1_-score were calculated separately for R00, R01, R03, R04, R05, R06, R07, and R09 for Llama 3.3-70B and gpt-oss-120B to assess variation in performance across individual cardiorespiratory code groups.

b *ICD-10-CM*: *International Classification of Diseases, Tenth Revision, Clinical Modification*.

## Discussion

### Principal Findings

We evaluated the ability of Llama 3.3-70B and gpt-oss-120B to extract S&S and map them to corresponding *ICD-10-CM* codes. All *ICD-10-CM* codes were strictly limited to the range R00 to R09 during the manual coding process. For this reason, providing the *ICD-10-CM* codes to the model led to better performance in the *ICD-10-CM* generation task compared to the S&S extraction task. Among the different prompting strategies tested, the multimodule LLMs with postdata cleaning achieved the highest performance across both tasks.

Initially, we only provided basic task instructions to test whether the general-purpose model possessed sufficient medical knowledge from its training data and whether it could correctly interpret medical terminology. The results showed that the model performed well in extracting medical terms. Table 6 presents sample output from 4 different prompt-engineering strategies. While the model was able to identify some S&S terms, it struggled to accurately recognize those related specifically to the cardiorespiratory system. It often failed to distinguish S&S from other medical terms and sometimes misclassified conditions as S&S. For example, the model incorrectly identified “peripheral vascular disease” as a symptom or sign, even though it is a diagnosis. Additionally, it mislabeled terms from other body systems, such as classifying “trace edema at dorsum of feet and ankles” as a cardiorespiratory symptom [14].

**Table 6.** Representative outputs generated by the large language model (LLM) under 4 prompt-engineering conditions (instruction-only, *ICD-10-CM* definition-based, assumption-free constraints, and multimodule LLM with postprocessing) for a single deidentified clinical note containing cardiorespiratory symptoms^a^.

Prompt setting	Sample output
Instruction only	“Here are the extracted symptoms and signs involving the circulatory and respiratory systems: **Circulatory System:** Ventricular ectopy (I47.1) - PVCs^b^ random, run off three beats consecutive History of coronary artery disease (I25.10) Peripheral vascular disease (I73.9) Hypertension (I10) **Respiratory System:** Cough (R05) Shortness of breath (R06.00) Decreased air entry (R09.02) COPD^c^ (J44.9) Note that I excluded denied or negative symptoms and signs, as per the requirement. Also, some codes may have additional characters to specify laterality or other details, but I provided the most relevant code based on the information given.”
*ICD-10-CM*^d^ code-based symptom extraction	“Here is the list of symptoms and signs involving the circulatory and respiratory systems along with their ICD-10 codes: * Cough - R05 * Shortness of breath - R06.0 (Dyspnea) * PVCs (Ventricular ectopy) - R00.1 (Bradycardia, unspecified is not correct as it’s actually ventricular ectopy which falls under other abnormalities of heart beat) - R00.8 * Tachycardia - R00.0 (The patient has a heart rate of 90) * Elevated blood-pressure reading - R03.0 Note: The ICD-10 codes are based on the provided list and may not be comprehensive or entirely accurate without further context or information.”
Assumption-free constraints	“Based on the clinical note, the patient’s current presented symptoms and signs involving the circulatory and respiratory systems are: Cough (R05) Shortness of breath (R06.0 Dyspnea) The following ICD-10 codes can be assigned: R05 (Cough) R06.0 (Dyspnea) Note: PVCs (Premature Ventricular Contractions) are mentioned in the note, but they do not have a direct ICD-10 code match from the provided list. However, it is related to the cardiovascular system and can be associated with abnormalities of heart beat, but since there’s no explicit mention of symptoms like palpitations or tachycardia that directly correlate with the PVCs as experienced by the patient, we cannot include R00 codes based on the given instructions.”
Multiodule LLMs with post data cleaning	
Extraction module	“Based on the provided definitions of symptom and sign, here are the patient’s current presented symptoms and signs extracted from the clinical note: **Symptoms:** Coughing episodes for about two weeks on and off Shortness of breath Neck pain Arthritis (patient is positive for arthritis) **Signs:** PVCs (premature ventricular contractions) random on monitor Decreased air entry in lungs S4 at the apex (heart sound) Cough (mentioned under REVIEW OF SYSTEMS: RESPIRATORY) Note that I have excluded: * Denied or negative symptoms and signs (e.g., no chest pain, no palpitations, etc.) * Signs with numerical data (e.g., heart rate of 90, blood pressure of 187/105, etc.) as per the requirement.”
Refinement module	R05: Coughing episodes for about two weeks on and off, R06.0: Shortness of breath, R00.1: PVCs (premature ventricular contractions), R01.1: S4 at the apex (heart sound), R06.8: Decreased air entry in lungs

a The examples illustrate differences in hallucination, inference, symptom specificity, and *ICD-10-CM *mapping accuracy. *ICD-10-CM* code evaluation in this study was performed at the 3-character category level; therefore, subcode-level differences were not counted as errors.

b PVC: premature ventricular contraction.

c COPD: chronic obstructive pulmonary disease.

d *ICD-10-CM*: *International Classification of Diseases, Tenth Revision, Clinical Modification*.

The Llama model could generate *ICD-10-CM* codes, suggesting that the coding system was included in its training data by Meta. However, hallucination remained a concern, as the model occasionally produced nonexistent codes. Moreover, some medical terms lacked clear boundaries between symptoms, signs, and diagnoses. In the *ICD-10-CM* system, the R00-R99 range is designated for S&S, but certain valid S&S still had to be mapped to codes outside this range. Due to our labeling strategy, which only included S&S mapped to R00-R09, the model’s performance appeared limited. Nonetheless, the model demonstrated its ability to extract medical concepts and associate them with *ICD-10-CM* codes, warranting further investigation.

We then provided a list of *ICD-10-CM* codes and their definitions related to the cardiorespiratory system with the prompt designed to minimize hallucinations and improve the model’s ability to identify relevant S&S. Model performance improved significantly and was able to extract most cardiorespiratory-related S&S accurately. Furthermore, the model exhibited the ability to draw inferences based on clinical context, which contributed to high recall. However, this introduced new challenges, including overinterpretation and assumption-based errors, resulting in lower precision. For instance, in one note that mentioned “congested respirations,” the model inferred and generated “cough” as a symptom, justifying it with the interpretation: “congested respirations and mild crackles are present, which can be related to cough (R05).” This example illustrates the model’s tendency to infer S&S based on contextual associations rather than relying solely on explicitly stated information, resulting in false positives.

To reduce overinterpretation, we refined our third prompt to instruct the model to extract only explicitly stated S&S. This adjustment improved precision, as a higher proportion of the identified terms was correct, while recall declined only slightly.

In the fourth prompt, we enforced a standardized output format that included only the extracted S&S along with their corresponding *ICD-10-CM* codes. This structured format was designed to support an automated evaluation pipeline. However, imposing all constraints within a single model introduced complexity. The model was expected to complete 6 distinct tasks simultaneously: extract S&S, restrict to the cardiorespiratory system, map to *ICD-10-CM* codes, exclude negated S&S, include only explicitly mentioned ones, and format the output accordingly. Relying on a single model led to occasional errors, such as missing explicitly mentioned S&S or including inferred ones, thereby reducing overall accuracy.

The multimodule LLM approach addressed this issue by distributing subtasks across specialized models, leading to more stable and accurate outputs. Additionally, by including definitions of S&S within the prompt, the model gained a clearer understanding of the extraction task. However, through further testing, we observed that the model often misclassified numerical examination results, such as blood pressure readings, as S&S. To address this, we explicitly instructed the model to exclude examination findings with numerical data. This comprehensive strategy resulted in the highest performance.

### Comparison With Prior Work

Prior work on clinical text analysis has largely focused on downstream prediction rather than explicit symptom extraction. Transformer-based models such as ClinicalBERT and Med-BERT have demonstrated strong performance in modeling clinical notes for outcomes such as hospital readmission and disease risk prediction; however, they do not explicitly extract S&S or map them to standardized coding systems such as *ICD-10-CM* [6,8,11,12]. Applying traditional machine learning models to our task would require large amounts of labeled training data for both named entity recognition and entity linking, substantially increasing data requirements, time investment, and labor burden.

More recent studies have applied LLMs to structured information extraction using prompt engineering. For example, prompt-based GPT approaches have been used to extract clinical factors from pathology or radiology reports with high accuracy and improved efficiency compared with manual abstraction [9]. However, these studies typically relied on proprietary, cloud-based models and did not systematically address hallucination or over-inference when processing narrative clinical notes. Hybrid retrieval-augmented generation pipelines have also been proposed to improve precision in extracting specific clinical attributes, such as substance use, by constraining model input to relevant text segments [13]. While effective, these approaches introduce additional system complexity and external dependencies.

In contrast, our study demonstrates that a locally deployed, open-source LLM, combined with assumption-free prompting and a multimodule framework, can accurately extract explicitly stated cardiorespiratory S&S and map them to *ICD-10-CM* codes. This work extends prior research by emphasizing hallucination control, standardized coding, and privacy-preserving deployment, highlighting the feasibility of open-source LLMs for reliable clinical symptom extraction. Future model development should adhere to principles of responsible artificial intelligence apps in medicine [24]. Accurate extraction of symptoms from clinical notes can facilitate artificial intelligence–assisted clinical decision support embedded in electronic health records [25,26].

### Limitations and Future Directions

Although the multimodule framework with postprocessing achieved the best overall performance, it did not fully eliminate subcode-level imprecision in *ICD-10-CM* mapping. For example, in Table 6, the refinement module mapped premature ventricular contractions to R00.1, which denotes unspecified bradycardia and is clinically discordant with premature ventricular contractions. This, therefore, represents a clinically incorrect mapping error. However, because our evaluation considered only the first 3 digits of the *ICD-10-CM* code, this output was treated as a correct R00 category-level mapping (abnormalities of heartbeat). Future work should improve subcode-level specificity and clinical precision in *ICD-10-CM* assignment.

The red-flag keyword filter used during postprocessing was intentionally conservative and improved overall performance, but it was also a coarse rule-based step. For example, because the keyword list included terms such as “explicitly,” valid outputs could theoretically be removed if the model echoed instruction-like language in its structured response. Although postprocessing improved overall *F*_1_-scores, this approach may have increased precision at the cost of removing some true positive outputs and should be refined in future work.

In addition, the scope of this study was restricted to cardiorespiratory S&S, which limits generalizability to other clinical domains. Future work should expand the dataset, incorporate a broader range of symptom domains, and further refine prompt engineering and multimodule orchestration strategies to improve robustness and adaptability across diverse biomedical contexts.

This study did not systematically evaluate model-specific runtime options, such as reasoning effort levels or structured output enforcement, because the primary objective was to assess the effects of prompt design and workflow structure under a fixed inference setting. We also did not compare our framework against proprietary frontier models, such as GPT-4. Although such models could provide an additional reference benchmark, they would not represent a fixed upper performance bound because extraction accuracy depends on factors such as model version, prompting strategy, runtime configuration, and evaluation design. In addition, this study was intentionally focused on privacy-preserving, locally deployable workflows for clinical text processing. Future work should investigate whether runtime configuration options further improve output stability and extraction accuracy and should include direct comparisons between open-weight local models and high-performing closed commercial models under standardized prompts and evaluation settings.

### Conclusions

In this study, Llama 3.3-70B was used primarily for prompt development to support the extraction of clinical S&S and their mapping to *ICD-10-CM* codes. We then evaluated the final multimodule LLM framework with postprocessing on an independent test set of 500 clinical notes using both Llama 3.3-70B and gpt-oss-120B. Under the entity-level microaveraged evaluation across the full corpus, Llama 3.3-70B achieved *F*_1_-scores of 0.73 for S&S extraction and 0.69 for *ICD-10-CM* code generation, whereas gpt-oss-120B achieved *F*_1_-scores of 0.88 and 0.87, respectively. In note-level macroaveraged evaluation, performance was higher, with Llama 3.3-70B achieving *F*_1_-scores of 0.95 for both S&S extraction and *ICD-10-CM* mapping and gpt-oss-120B achieving *F*_1_-scores of 0.97 and 0.98. Overall, the framework demonstrates the feasibility of locally deployable LLMs for structured extraction of cardiorespiratory S&S from clinical notes and may increase the level of privacy safety by allowing on-premises processing without external data transmission.

## Abbreviations

COPD: chronic obstructive pulmonary disease
EM: extraction module
*ICD-10-CM*: *International Classification of Diseases, Tenth Revision, Clinical Modification*
LLM: large language model
NLP: natural language processing
RM: refinement module
S&S: symptom and sign
